# Sinapic Acid Attenuates Chronic DSS-Induced Intestinal Fibrosis in C57BL/6J Mice by Modulating NLRP3 Inflammasome Activation and the Autophagy Pathway

**DOI:** 10.1021/acsomega.3c07474

**Published:** 2023-12-26

**Authors:** Wan-Ying Li, Jun-Yang Liu, Zi-Xian Wang, Ke-Ying Wang, Chun-Xiang Huang, Wen He, Jia-Le Song

**Affiliations:** † Department of Nutrition and Food Hygiene, School of Public Health, Guilin Medical University, Guilin 541100, Guangxi, China; ‡ Department of Clinical Nutrition, Liuzhou People’s Hospital, Liuzhou 545006, Guangxi, China; § Guangxi Key Laboratory of Environmental Exposureomics and Entire Lifecycle Health, Guilin Medical University, Guilin 541100, Guangxi, China; ∥ Department of Clinical Nutrition and Obstetrics, The Second Affiliated Hospital of Guilin Medical University, Guilin 541199, Guangxi, China

## Abstract

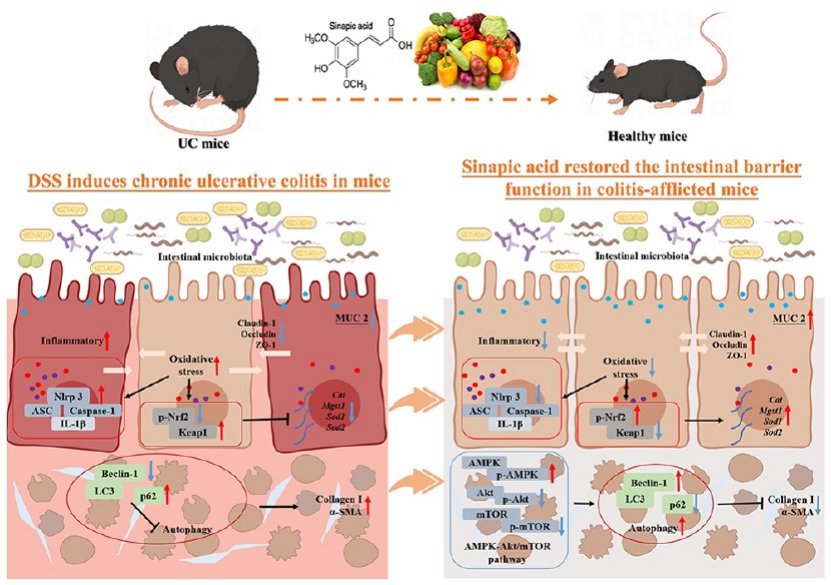

Ulcerative colitis (UC) is a chronic gastrointestinal disease that results from repeated inflammation and serious complications. Sinapic acid (SA) is a hydroxycinnamic acid present in a variety of plants that has antioxidant, anti-inflammatory, anticancer, and other protective effects. This study investigated the antifibrotic effect of SA on chronic colitis induced by dextran sulfate sodium salt (DSS) in mice. We observed that SA could significantly reduce clinical symptoms (such as improved body weight loss, increased colon length, and decreased disease activity index score) and pathological changes in mice with chronic colitis. SA supplementation has been demonstrated to repair intestinal mucosal barrier function and maintain epithelial homeostasis by inhibiting activation of the NLRP3 inflammasome and decreasing the expression of IL-6, TNF-α, IL-17A, IL-18, and IL-1β. Furthermore, SA could induce the expression of antioxidant enzymes (*Cat*, *Sod1*, *Sod2*, *Mgst1*) by activating the Nrf2/keap1 pathway, thus improving antioxidant capacity. Additionally, SA could increase the protein expression of downstream LC3-II/LC3-I and Beclin1 and induce autophagy by regulating the AMPK-Akt/mTOR signaling pathway, thereby reducing the production of intestinal fibrosis-associated proteins Collagen-I and α-SMA. These findings suggest that SA can enhance intestinal antioxidant enzymes, reduce oxidative stress, expedite intestinal epithelial repair, and promote autophagy, thereby ameliorating DSS-induced colitis and intestinal fibrosis.

## 1 Introduction

Ulcerative colitis (UC) is a subtype of inflammatory bowel disease (IBD), which is a chronic nonspecific inflammatory condition that primarily affects the colon and rectum. An estimated 1/1000 people worldwide suffer from IBD, and the incidence of IBD in China is increasing.^1−3^ People with UC often have abdominal discomfort and blood in their stools, and the long-term course of the disease can lead to complications and even colorectal cancer.^4^ Current treatments such as mesalazine and dexamethasone may have potential side effects such as steroid dependence and serious infections and are not always effective in preventing and treating UC.^5^ Thus, it is important to find an effective and safe treatment for UC.

The NLRP3 inflammasome, which is composed of the NLRP3 scaffold, an apoptosis-associated specklike protein containing a CARD (ASC), and an effector protein cysteine protease 1 precursor (pro-caspase-1), plays a crucial role in the development of intestinal inflammation and the resultant intestinal barrier damage.^6,7^ NLRP3 is initially activated by external stimuli, recruits ASC proteins and pro-caspase-1, and increases the production of IL-1β and IL-18, thus accelerating epithelial damage and intestinal inflammation.^8^

Reactive oxygen species (ROS) are highly active molecules, including a superoxide anion (O^2–^), hydrogen peroxide (H_2_O_2_), and the hydroxyl radical (OH^–^). ROS are produced by the normal metabolism of the body under physiological conditions. However, when excessive ROS are produced, they can lead to endoplasmic reticulum stress and cause various adverse biological reactions, resulting in the occurrence of various diseases.^9,10^ Excessive production of ROS by infiltrated neutrophils during chronic intestinal inflammation can induce oxidative stress, resulting in cellular damage, which in turn promotes further inflammatory cell infiltration and can lead to intestinal mucosal ulceration and necrosis.^11^ Nuclear factor E2 related factor 2 (Nrf2) is the gene that controls the antioxidant reaction element (ARE).^12^ In response to stimulation by ROS and inflammatory cytokines, Nrf2 separates from Keap1 and subsequently translocates to the nucleus, thereby activating the expression of endogenous antioxidant factors such as GSH-Px, SOD, and CAT, decreasing the expression of inflammatory cytokines and improving experimental colitis.^13^

Autophagy is a physiological process prevalent in eukaryotic cells that involves the phagocytosis of its own cytoplasmic proteins or organelles and their encapsulation into vesicles followed by their fusion with lysosomes to form autophagic lysosomes. These lysosomes then digest and degrade the encapsulated contents, thus regulating the metabolism and renewal of the cells.^14^ First, autophagic vesicles are formed through the autophagy-associated gene UKL1 and Beclin 1 protein complex, and then the autophagic vesicles bind with autophagy-associated proteins and microtubule-associated protein light chain 3-II (LC3-II) to form autophagosomes, which capture proteins, organoids, and other substances that need to be degraded.^15^ Finally, autophagosomes and lysosomes fuse to release their contents and complete autophagy. Microtubule-associated protein light chain 3-I (LC3-I) is catalyzed by the enzymes Atg7 and Atg3 to form the autophagosome marker LC3-II during autophagy.^16^ High levels of LC3-II synthesis can confirm the formation of autophagosomes and suggest an increase in autophagic activity.^17^ Conversely, the adaptor protein p62 can bind to ubiquitinated proteins and organelles and, in response to the binding of LC3-II, transport them into the autophagosome for degradation.^15^ Therefore, LC3, Beclin1, and p62 are important factors in autophagy and are often used as indicators to judge the level of autophagy. The adenylate-activated protein kinase (AMPK)-protein kinase B (PKB/Akt)/mammalian rapamycin target (mTOR) pathway is the most classic autophagy signaling pathway. When AMPK is phosphorylated, the activity of mTOR is reduced, which triggers the initiation of the autophagy cascade.^18^ PKB/Akt is a threonine protein kinase that works with phosphatidylinositol-dependent protein kinase 1/2 to promote the binding of phosphatidylinositol triphosphate to itself. Akt is transferred from the cytoplasm to the plasma membrane and phosphorylated. The activation of Akt activates the downstream protein mTOR.^19^ Autophagy dysregulation causes intestinal epithelial damage, compromising epithelial barrier integrity and the mucosal immune response.^20^

Sinapic acid (5-dimethoxy-4-hydroxycinnamic acid, SA), which is a simple phenolic compound rich in vegetables, grains, and citrus fruits,^21^ has been reported to have various pharmacological activities, such as antioxidant, anti-inflammatory, anticancer, and antiobesity activities.^22−26^ On the other hand, SA has strong activity against bleomycin-induced pulmonary fibrosis in rats,^27^ attenuates chemical reagent (such as 2,4,6-trinitrobenzenesulfonic acid, TNBS, and DSS)-induced clinical signs, and alleviates colonic inflammation in a rodent model of colitis.^23,28^ In addition, our previous study suggested that SA inhibited inflammation-induced intercellular hyperpermeability and maintained epithelial homeostasis in lipopolysaccharide-induced Caco-2 cells.^22^ During long-term chronic intestinal inflammation, excessive production of collagen can occur, and excessive accumulation of collagen in the intestine can lead to colon fibrosis.^29^ The aim of this study was to examine the effect and mechanism of SA treatment in alleviating colonic fibrosis caused by chronic colitis using DSS-induced chronic colitis model mice.

## 2 Materials and Methods

### 2.1 Chemical Reagents

SA and 5-aminosalicylic acid (5ASA) were purchased from Aladdin Chemicals Co., Ltd. (Shanghai, China). The standard AIN-93G diet was purchased from SYSE Biotechnology Co., Ltd. (Changzhou, China). DSS was obtained from MP Biomedicals (Santa Ana, USA). Carboxymethyl cellulose (CMC), the H&E staining kit, Masson’s trichrome staining solution, and the primary antibodies (Caspase1, LC3, β-actin) were acquired from Servicebio Biotechnology Co., Ltd. (Wuhan, China). Anti-ASC (AF6234), anti-α-SMA (AF1507), anti-Beclin1 (AF5123), anti-p62 (AF5312), anti-Nrf2 (AF7623), anti-p-Nrf2 (AF1609), anti-Keap1 (AF7335), anti-AMPK (AF1627), anti-p-AMPK (AF2677), anti-Akt (AF1777), anti-p-Akt (AF1546), and anti-mTOR (AF1648) antibodies were acquired from Beyotime Biotechnology Co., Ltd. (Shanghai, China). Anti-NLRP3 (ab263899), anticollagen-I (ab270993), and anti-p-mTOR (ab109268) were obtained from Abcam (Cambridge, MA, USA). Anti-IL-1β (PTR2541) was acquired from ImmunoWay Biotechnology Co., Ltd. (Jiangsu, China).

### 2.2 Experimental Animals and Study Design

Six-week-old C57BL/6J mice (male, 18–22 g) were obtained from Hunan SJL Animal Co., Ltd. (Changsha, China). The animals had ready access to standard water and AIN-93G food and were housed in a standard SPF environment (12/12 h cycle, 24 ± 2 °C). After 7 days of adaptation, the mice were randomly divided into the normal, DSS, SA (40 and 80 mg/kg), and 5ASA (50 mg/kg) groups. To establish a chronic colitis rodent model, the mice were first given a 2.5% DSS solution (w/v) for 5 days, which was then changed to sterile water for 2 days, and this cycle was repeated 4 times for a total of 28 days according to the study of Li et al.^30^ On the 15th to 28th day, the mice were intragastrically administered SA and 5ASA (SA and 5ASA dissolved in 1% CMC), while mice in the DSS group received 1% CMC. The normal group was fed routinely for 28 days. Body weights were measured every 3 days during the experiment. The length and weight of the colon were quickly estimated. The Institutional Animal Care and Use Committee of Guilin Medical College approved the experimental protocols (review code: GLMC-IACUC-2020027).

### 2.3 Disease Activity index (DAI)

Body weight, blood loss, and fecal composition were recorded every three days during the experiment. The DAI score was evaluated to determine the degree of colon inflammation and was calculated according to the methodology reported by Khan et al.^23^

### 2.4 Serum Preparation and ELISA Analysis

The mice were first anesthetized by an injection of 1.25% tribromoethanol, and their blood was collected through the inferior vena cava and centrifuged in a Beckman Coulter X-22R centrifuge (3000 rpm, 4 °C, 10 min) to prepare serum samples. The levels of tumor necrosis factor (TNF-α, MM-0132M2), IL-1β (MM-0040M2), IL-6 (MM-0163M2), IL-17A (MM-0759M2), and IL-18 (MM-0169M1) in the serum were then determined using the reagents and instructions provided by Jiangsu Meimian Industrial Co., Ltd. (Yancheng, China).

### 2.5 Histopathological Analysis

After executing mice, the colon weight and length were measured. H&E and Masson staining of the colon tissue were performed according to our previous studies,^23^ and the histological observation using a DM4B microscope (Leica Microsystems, Buffalo Grove, USA) was recorded.

### 2.6 Quantitative Real-Time Quantitative PCR (qRT–PCR) Assay

According to our previous study,^23^ total RNA was obtained from mouse colon tissue according with the TRIzol reagent, and the absorbance was measured to determine the purity and concentration. cDNA was obtained by reverse transcription. The primers (Table 1) were mixed with the cDNA, and a quantitative PCR kit (Tiangen) was used to detect NLRP3 inflammasome factors (*Nlrp3*, *Caspase1*, *Asc*, and *Il1β*), fibrotic factors (alpha-smooth muscle actin, *αSma*, and *CollagenI*), and antioxidant enzymes (*Cat*, *Sod1*, *Sod2*, and *Mgst1*) using a real-time PCR system. The mRNA level of *Gapdh* was measured as an internal control.

**Table 1:** Gene Primers Used in This Study

Gene name	Primer sequence (5′-3′)	Primer sequence (3′-5′)
*Nlrp3*	CCGTCTACGTCTTCTTCCTTTC	CGCAGATCACACTCCTCAAATA
*Asc*	GTTCAAGATGAAGCTGCTGAC	TTCTGGCTGTGCCCTGAGCA
*Caspase1*	TACACGTCTTGCCCTCATTATC	CTCCAGCAGCAACTTCATTTC
*Il1β*	GGTACATCAGCACCTCACAA	TTAGAAACAGTCCAGCCCATAC
*CollagenI*	AGACCTGTGTGTTCCCTACT	GAATCCATCGGTCATGCTCTC
*α-SMA*	CCCAGACATCAGGGAGTAATGG	TCTATCGGATACTTCAGCGTCA
*Cat*	GATGGTAACTGGGATCTTGTGG	GTGGGTTTCTCTTCTGGCTATG
*Sod1*	CGGATGAAGAGAGGCATGTT	TAGTACGGCCAATGATGGAATG
*Sod2*	AGCGTGACTTTGGGTCTTT	AGCGACCTTGCTCCTTATTG
*Mgst1*	AGCCCACCTGAATGATCTTG	GTAGATCCGAGCACCTACAAAG
*Gapdh*	GGAGAAACCTGCCAAGTATGA	TCCTCAGTGTAGCCCAAGA

### 2.7 Western Blot Analysis

Western blot analysis was performed according to our previously studies.^31^ The total protein was rapidly extracted from colon tissue (30 mg) with lysis buffer, ground in a KZ-II high-speed tissue homogenizer, and centrifuged (10000 rpm, 4 °C) for 10 min. Protein samples were obtained, and their concentrations were determined by using a BCA kit. The protein samples were separated on 10%–12% SDS–PAGE gels, transferred to 0.45 μm of poly(vinylidene fluoride) membranes, and then blocked with 5% skim milk or BSA in TBST. After that, the membranes were incubated overnight with the indicated primary antibodies at 4 °C. After the primary antibody incubation was completed, the membranes were washed with TBS-T buffer, incubated with horseradish peroxidase (HRP)-labeled sheep antirabbit IgG secondary antibodies for 1 h, and washed 3 times. The bands were visualized using an enhanced chemiluminescence solution (ECL) and examined by Fluor Chem MSystems (Protein Simple, California, USA). The density of the bands was analyzed using ImageJ (NIH, USA, https://imagej.net).

### 2.8 Periodic Acid-Schiff (PAS) and Alcian Blue (AB) Staining

After dewaxing and hydration, paraffin sections of mouse colon tissue were soaked and washed with distilled water (dH_2_O). Sections were stained with periodic acid oxidation solution and acidification solution, washed with anhydrous ethanol and xylene, and finally sealed with neutral gum.^32^ Finally, images were observed under a Leica microscope with a digital camera to examine the expression levels.

### 2.9 Immunohistochemistry

Immunohistochemical analyses of Claudin-1, ZO-1, and Occludin were performed on colonic tissues according to our previous studies.^31^ The paraffin sections were soaked twice with xylene and then hydrated with varying concentrations of ethanol and dH_2_O. Antigen retrieval was performed with EDTA antigen retrieval solution for 15 min. The sections were soaked in 3% H_2_O_2_ for 20 min to block endogenous peroxidase activity, incubated with 10% goat serum for 30 min, and then incubated with specific antibodies overnight at 4 °C. After incubation with the primary antibodies, the sections were then incubated with streptavidin-horseradish peroxidase for 20 min. Diaminobenzidine (DAB) chromogen was added and incubated at room temperature for 10 min. The sections were counterstained with hematoxylin and mounted on coverslips. Finally, images were acquired and recorded using a microscope (Leica).

### 2.10 Statistical Analysis

The data are presented as the mean ± standard deviation and were analyzed by using SPSS 25 (SPSS Inc., Chicago, IL, USA). To determine significance between groups, one-way analysis of variance (ANOVA) and Duncan’s multiple range tests were performed. A value of *p* < 0.05 was considered statistically significant.

## 3 Results

### 3.1 SA Improves Clinical Symptoms of Chronic Colitis Caused by DSS

**Figure 1:**
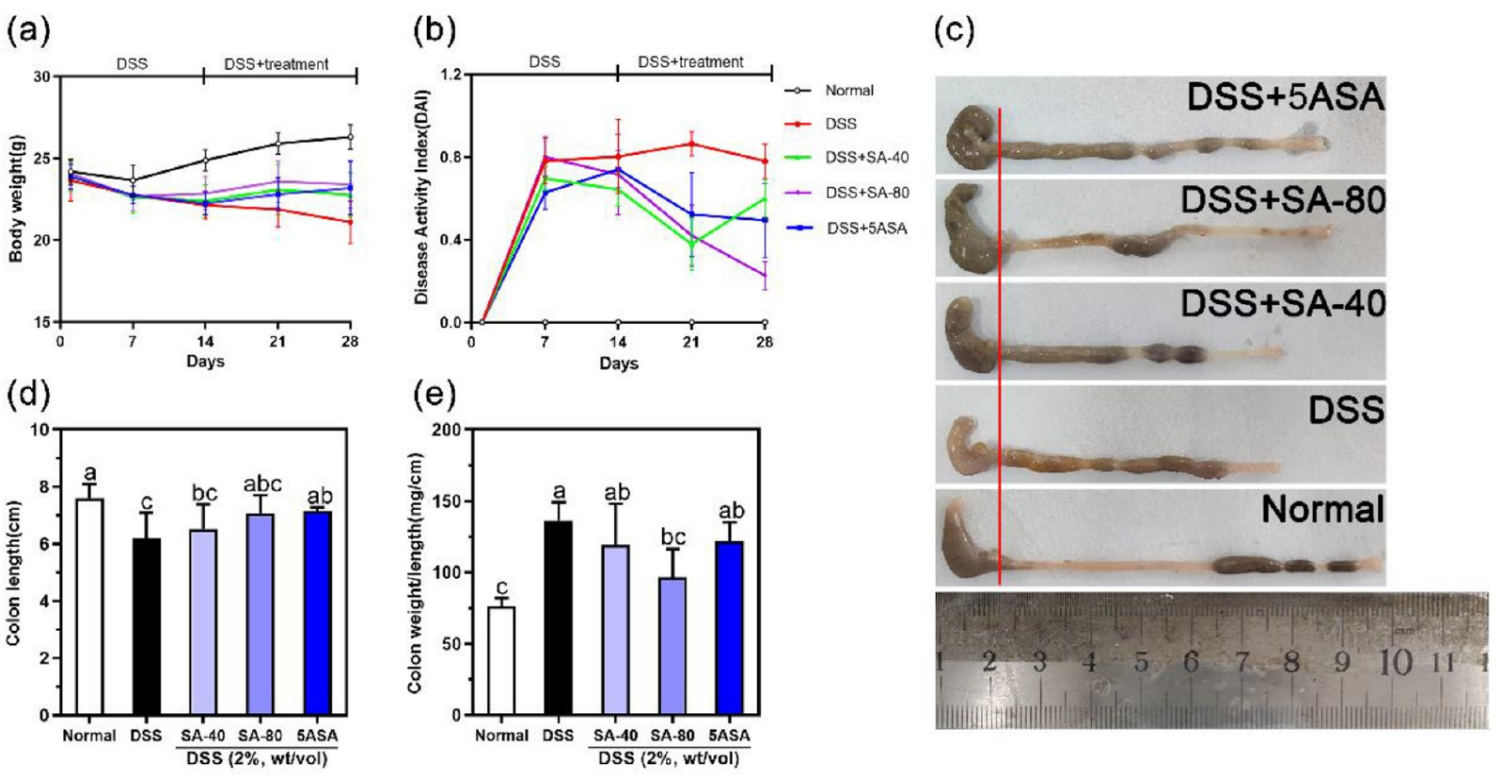
Sinapic acid (SA) ameliorates DSS-induced body weight loss: DAI score increases, colon shortening, and colon weight to length ratio in mice. (a) Body weight, (b) DAI score, (c, d) colon length, and (e) colon weight/length. Data were expressed as mean ± SD; *n* = 6. The letters above the bar indicate significant differences between the two groups (Duncan’s multiple range test; *p* < 0.05).

The common symptoms of colitis in mice are weight loss, diarrhea, and blood in the stools.^28^ On Days 14 and 28 of drug treatment, DSS-induced mice showed significant weight loss, and the body weight was visibly lower than that of the normal group. SA intervention ameliorated weight loss in the mice (Figure 1(a)). As shown in Figure 1b, the DAI scores increased significantly after DSS intervention, which was inhibited by SA treatment. Furthermore, DSS treatment shortened the colon length and thickened the colon wall compared with normal mice, while oral administration of SA significantly ameliorated these changes (Figure 1(c)–(e)).

### 3.2 SA Improves Colonic Histopathological Symptoms Caused by DSS

**Figure 2:**
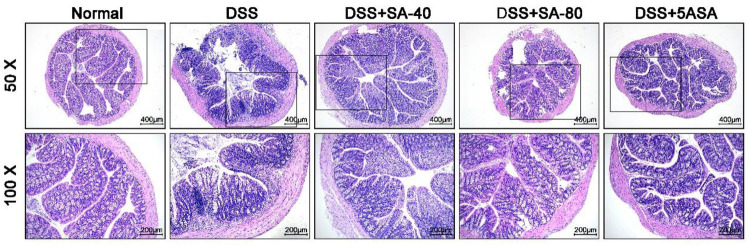
Effect of sinapic acid (SA) on colonic pathological changes in DSS-induced colitis mice. H&E staining (ruler 400 μm, black box area 200 μm).

HE staining showed that there was no colonic mucosal epithelial injury in normally fed mice. In contrast, DSS-induced colon damage in mice, including epithelial damage, inflammatory cell infiltration, and the loss of crypt structure, resulted in extensive histopathological changes. However, SA (40 and 80 mg/kg) repaired intestinal damage, restored crypt structure, improved mucosal damage, and reduced inflammatory cell infiltration, and 80 mg/kg of SA had the same effect as 5ASA in the positive normal group (Figure 2).

### 3.3 SA Improved Serum Overexpression of Proinflammatory Factors Caused by DSS

**Figure 3:**
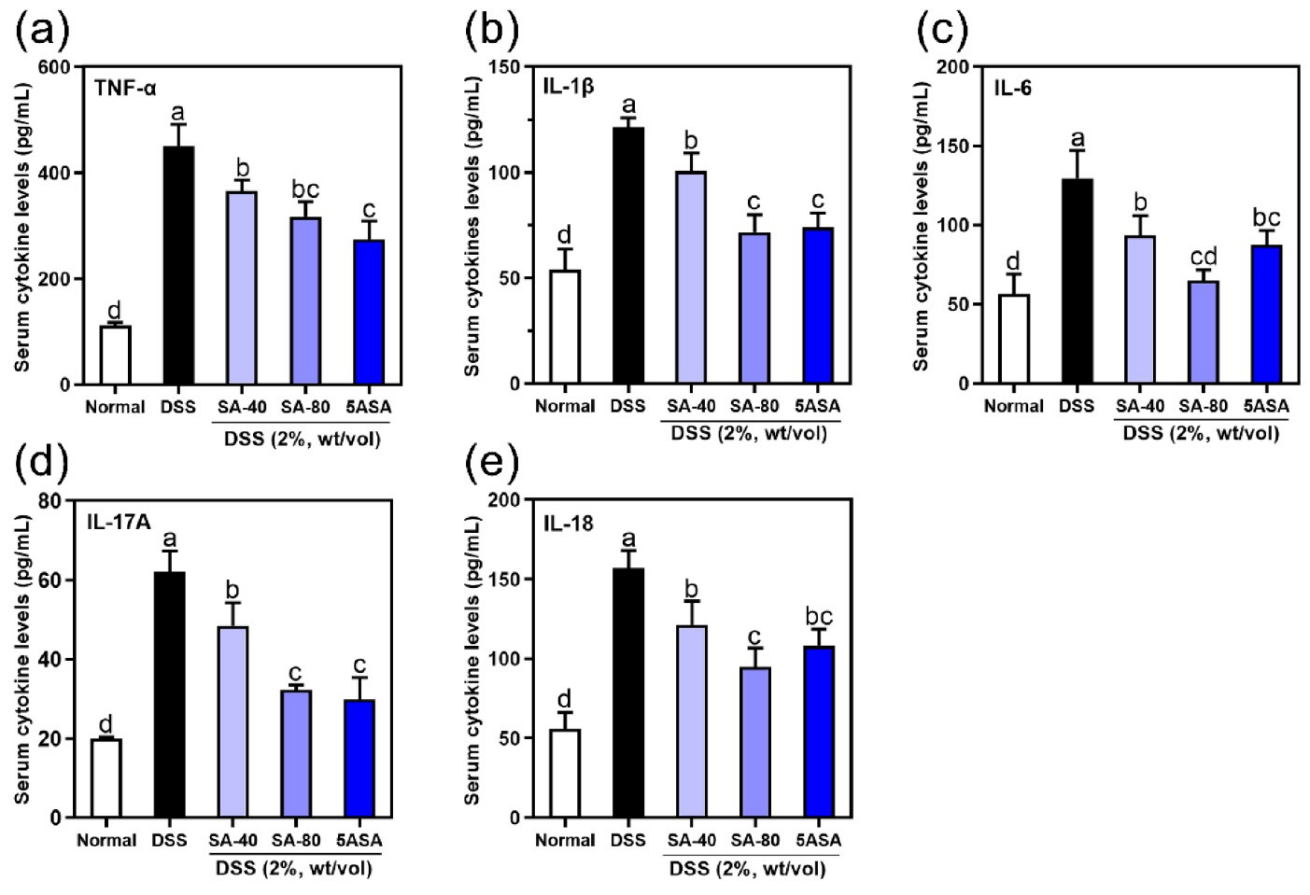
Effects of sinapic acid (SA) on serum inflammatory factors in DSS-induced colitis mice: (a) TNF-α, (b) IL-1β, (c) IL-6, (d) IL-17A, and (e) IL-18. Data were expressed as mean ± SD; *n* = 6. The letters above the bar indicate significant differences between the two groups (Duncan’s multiple range test; *p* < 0.05).

Proinflammatory factors are known to promote the development of colitis. DSS significantly increased the serum levels of IL-1β, TNF-α, IL-6, IL-17A, and IL-18 (*p* < 0.05). SA and 5ASA treatment visibly reduced the serum levels of IL-1β, IL-6, TNF-α, IL-17A, and IL-18 (*p* < 0.05). Higher doses of SA (80 mg/kg) were more effective in reducing the serum levels of IL-1β (41.12%), TNF-α (29.66%), IL-6 (50.09%), IL-17A (48.20%), and IL-18 (39.47%) in mice induced with DSS (*p* < 0.05, Figure 3(a)–(e)).

### 3.4 SA Improved the DSS-Induced Abnormal Protein and mRNA Expression of NLRP3 Inflammasome-Related Factors in the Colon

**Figure 4:**
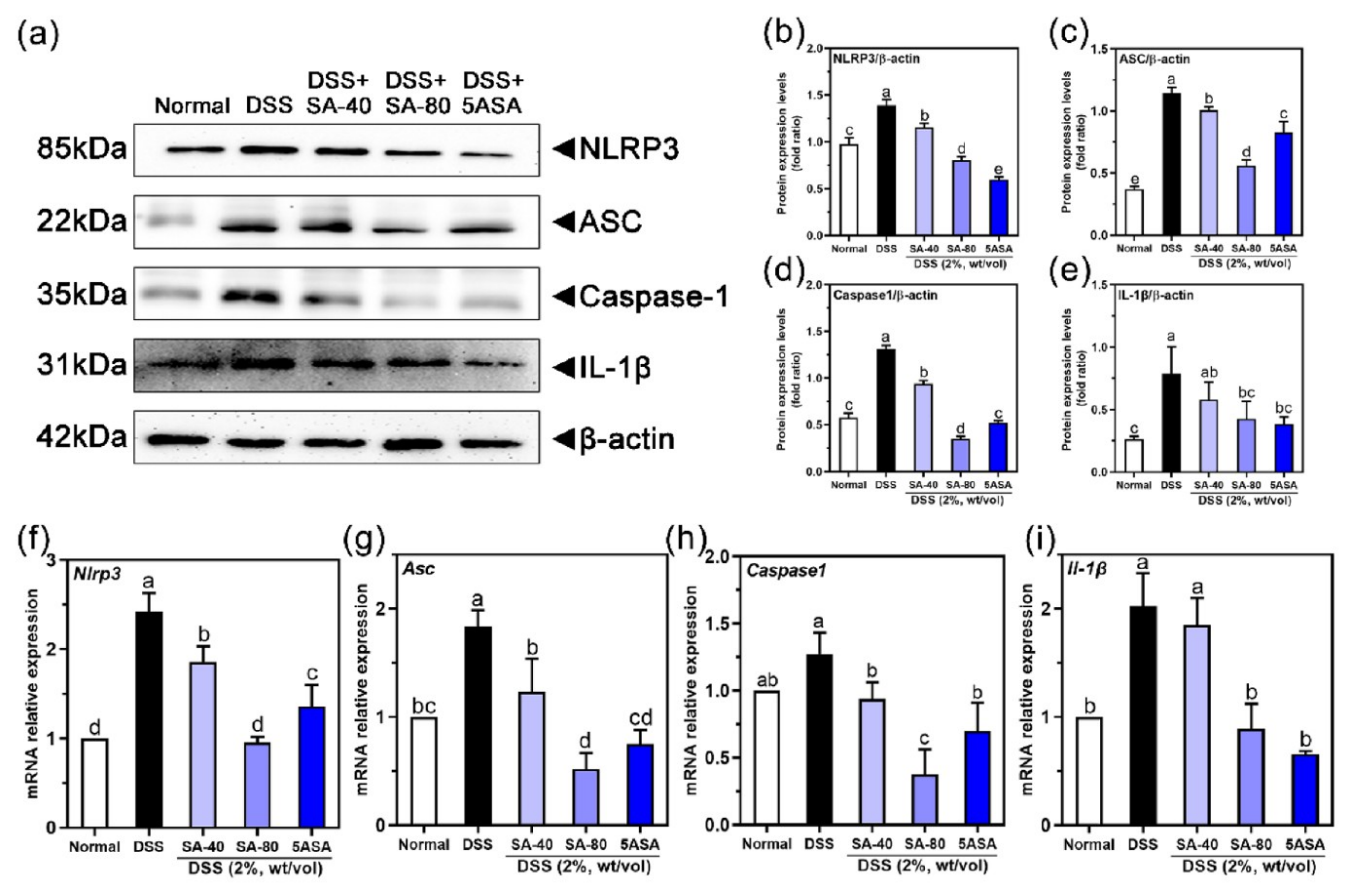
Effects of sinapic acid (SA) on proteins and mRNA of NLRP3 inflammasome in the colon of DSS-induced colitis mice: (a) protein Western blot detection of NLRP3, ASC, Caspase-1, and IL-1β protein expression; (b) NLRP3 protein expression; (c) ASC protein expression; (d) Caspase-1 protein expression, (e) IL-1β protein expression; (f) mRNA expression of Nlrp3; (g) mRNA expression of Asc; (h) mRNA expression of Caspase-1; and (i) mRNA expression of Il-1β. The letters above the bar indicate significant differences between the two groups (Duncan’s multiple range test; *p* < 0.05).

Figure 4(a)–(e) shows that compared to those in normally fed mice the protein levels of NLRP3 (42%), ASC (∼2.1 times), Caspase-1 (∼1.27 times), and IL-1β (∼2.01 times) in DSS-treated mice were increased (*p* < 0.05). Furthermore, the protein levels of NLRP, ASC, Caspase-1, and IL-1β were decreased after SA intervention (42%, 51.2%, 73.3%, and 46%, *p* < 0.05). Similarly, after SA intervention, the effects on gene expression were the same, as shown in Figure 4(f)–(i). Treatment with 80 mg/kg of SA downregulated the DSS-induced increase in the gene expression of *Nlrp3*, *Asc*, *Caspase1*, and *Il1β* (40.78%, 71.53%, 70.34%, and 56.11%, respectively) (*p* < 0.05).

### 3.5 SA Ameliorates Oxidative Stress in DSS-Induced Chronic Colitis Mice by Activating Nrf2

**Figure 5:**
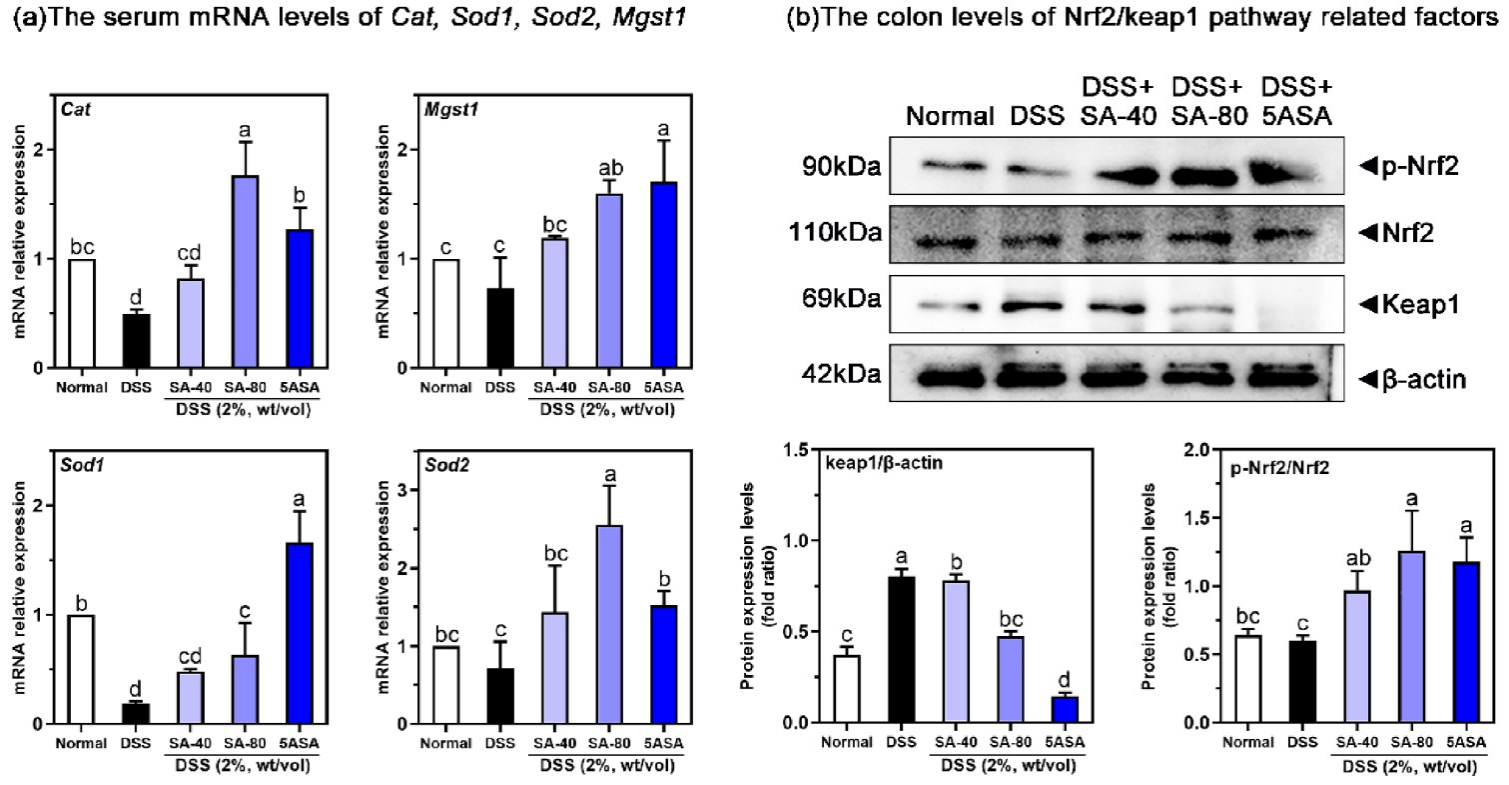
Effects of sinapic acid (SA) on the mRNA levels of serum antioxidant enzymes (a) and Nrf2/Keap1 protein (b) in colonic tissues of mice with colitis. qRT-PCR, using β-actin as a control, normalized the relative mRNA levels of each gene to those of the normal group. The letters above the bar indicate significant differences between the two groups (Duncan’s multiple range test; *p* < 0.05).

Figure 5(a) shows that SA-treated mice had greatly increased antioxidant enzyme levels in the colon tissues. The DSS group showed significantly elevated levels of *Cat* (51%), *Mgst1* (26.57%), *Sod1* (81.37%), and *Sod2* (28.47%) mRNA compared to the normal group (*p* < 0.05). After intervention with 40 mg/kg and 80 mg/kg of SA, the mRNA levels increased, and the effect of 80 mg/kg of SA was more obvious, resulting in increases of 260.54%, 118.25%, 240.43%, and 257.41%, respectively, which was similar to that of the 5ASA group (*p* < 0.05). Figure 5(b) shows the effect of SA on the Nrf2/Keap1 pathway upstream of antioxidant enzymes, as examined by Western blotting. DSS-induced mice had increased colonic protein levels of Keap1 (∼1.15 times) and decreased the protein levels of p-Nrf2 (6.2%) compared to normal mice (*p* < 0.05). However, 80 mg/kg of SA decreased the protein expression of Keap1 (40.8%, *p* < 0.05) and increased the protein level of p-Nrf2 (109.8%, *p* < 0.05). Similarly, 5ASA treatment had the same effect, upregulating the protein expression of p-Nrf2 (96.5%) and downregulating the protein expression of Keap1 (82%, *p* < 0.05).

### 3.6 SA Restored the Intestinal Barrier Structure and Mucosal Injury Induced by DSS in Chronic Colitis Mice

**Figure 6:**
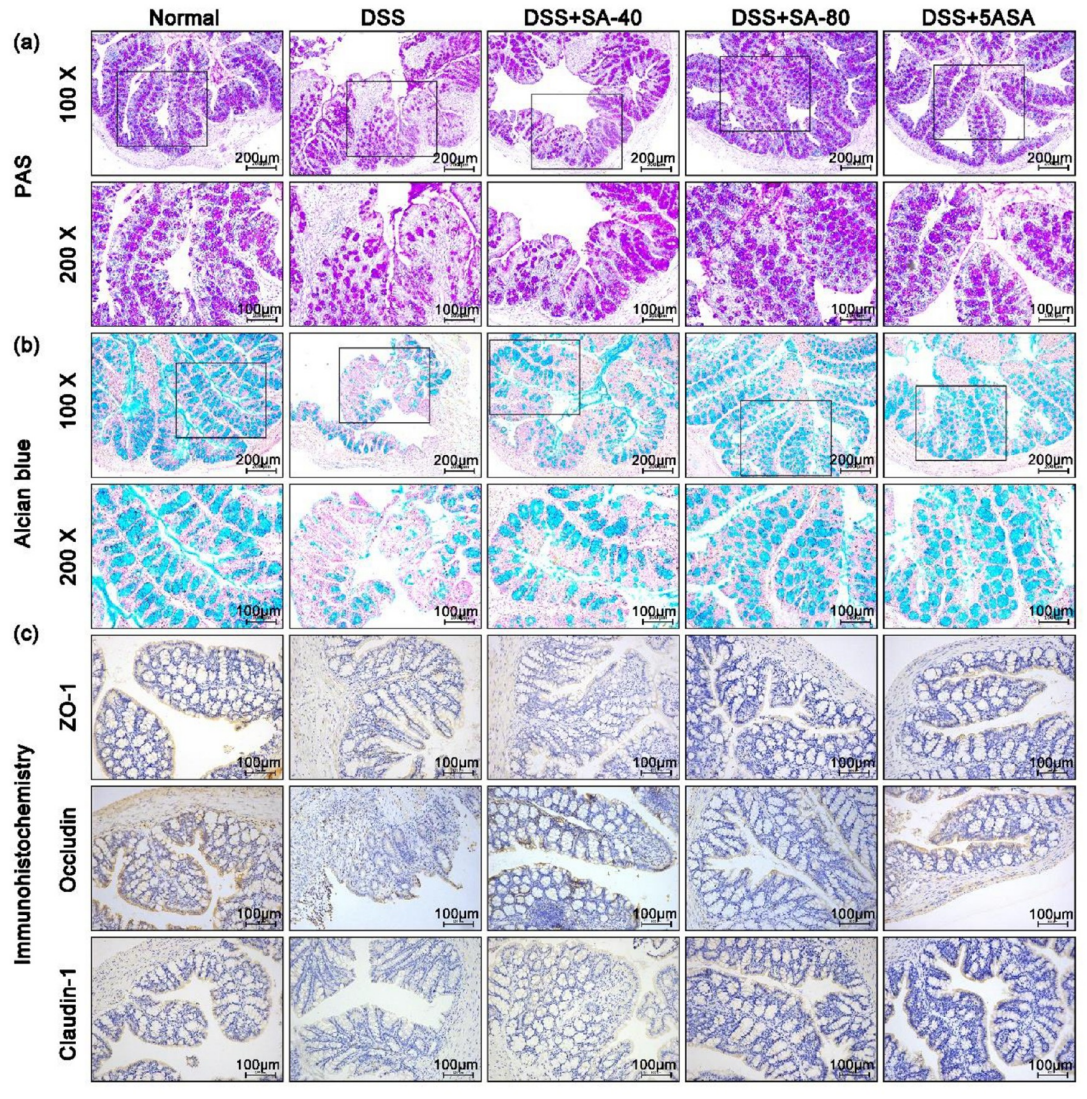
Effects of sinapic acid (SA) on intestinal mucosal injury and intestinal barrier function in DSS-induced chronic colitis mice. (a) Periodate Acid Schiff (PAS) staining was used to observe the distribution of colonic goblet cells in each group (ruler 200 μm, black box area 100 μm); (b) Alcian Blue staining was used to observe the secretion of colonic mucin in each group (ruler 200 μm, black box area 100 μm); and (c) immunohistochemistry was used to detect ZO-1, Occludin, and Claudin-1 proteins in the colon (ruler 100 μm).

As shown in Figure 6(a), PAS staining label goblet cells of mouse colon tissue were purple, and the number of goblet cells by DSS-treated mice was lower than that in normal mice. After 40 and 80 mg/kg of SA treatment, the number of goblet cells was more than that in the DSS group. Alcian blue staining can directly reflect the synthesis and secretion of MCU2 in mouse colon tissue. Figure 6(b) shows that colonic secretion and synthesis of mucin in DSS mice were significantly lower than those in the normal group. Importantly, SA treatment significantly increased mucin secretion and synthesis in colon tissues. We further detected the expression of Claudin-1, ZO-1, and Occludin. In addition, Figure 6(c) also shows that oral administration of SA and 5ASA significantly inhibited the loss of Claudin-1, ZO-1, and Occludin caused by DSS, indicating that SA could repair intestinal barrier damage, and the therapeutic effect of 80 mg/kg of SA was better than that of 40 mg/kg SA.

### 3.7 Effects of SA on Intestinal Fibrosis in Chronic Colitis Mice

**Figure 7:**
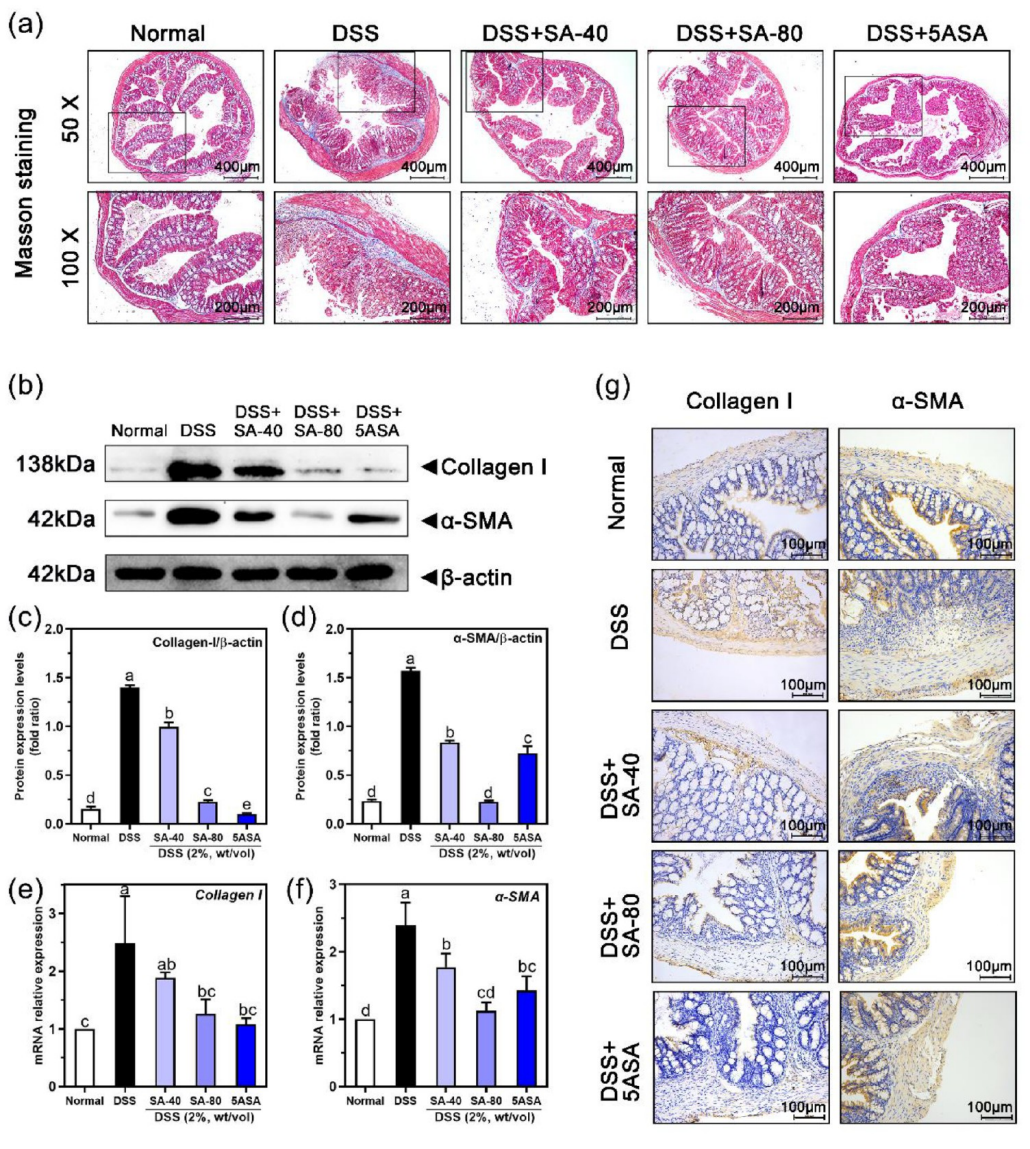
Effects of sinapic acid (SA) on intestinal fibrosis in DSS-induced chronic colitis mice. (a) Masson trichromatic staining of colon tissue (bar 400 μm, black box area 200 μm). (b) Protein expression of collagen-I and α-SMA was detected by Western blot. (c) Collagen-I protein expression. (d) α-SMA protein expression. (e, f) qRT-PCR was used to detect the mRNA expression levels of collagen-I and α-SMA in colon. Using β-actin as a control, the relative mRNA levels of each gene were normalized to those of the normal group; (g) Immunohistochemistry was used to detect α-SMA and collagen-I proteins in the colon (ruler 100 μm). Letters above the bar indicate significant differences between the two groups (Duncan’s multiple range test; *p* < 0.05).

Figure 7(a) shows the effect of SA on colonic fibrosis in mice with chronic colitis, and the degree of colonic fibrosis was detected using Masson’s triple staining. The DSS group showed significantly higher levels of colon fibrosis than the normal group, indicating severe collagen deposition. However, colon fibrosis was significantly inhibited in mice treated with SA and 5ASA compared with DSS-induced mice. The improvement in the 80 mg/kg SA group was more obvious. To further confirm whether SA inhibited the expression of α-SMA and collagen-I, we examined their expression by immunohistochemistry and Western blotting. The Western blot results in Figure 7(b)–(d) show that intestinal protein levels of α-SMA (∼5.7 times) and collagen-I (∼8.05 times) in colitis tissues were markedly increased in the DSS group, and the levels were substantially higher than those in the normal group (*p* < 0.05). However, similar to 5ASA-fed mice, the expression of α-SMA and collagen-I in colitis tissues in 40 mg/kg SA-treated mice (47% and 28.4%) and 80 mg/kg SA-treated mice (85.6% and 83.7%) was appreciably lower than that in DSS-induced mice (*p* < 0.05). Moreover, the qRT–PCR (Figure 7(e)–(f)) and immunohistochemistry (Figure 7(g)) results were similar to those described above.

### 3.8 SA Enhances Autophagy in Chronic Colitis Mice

**Figure 8:**
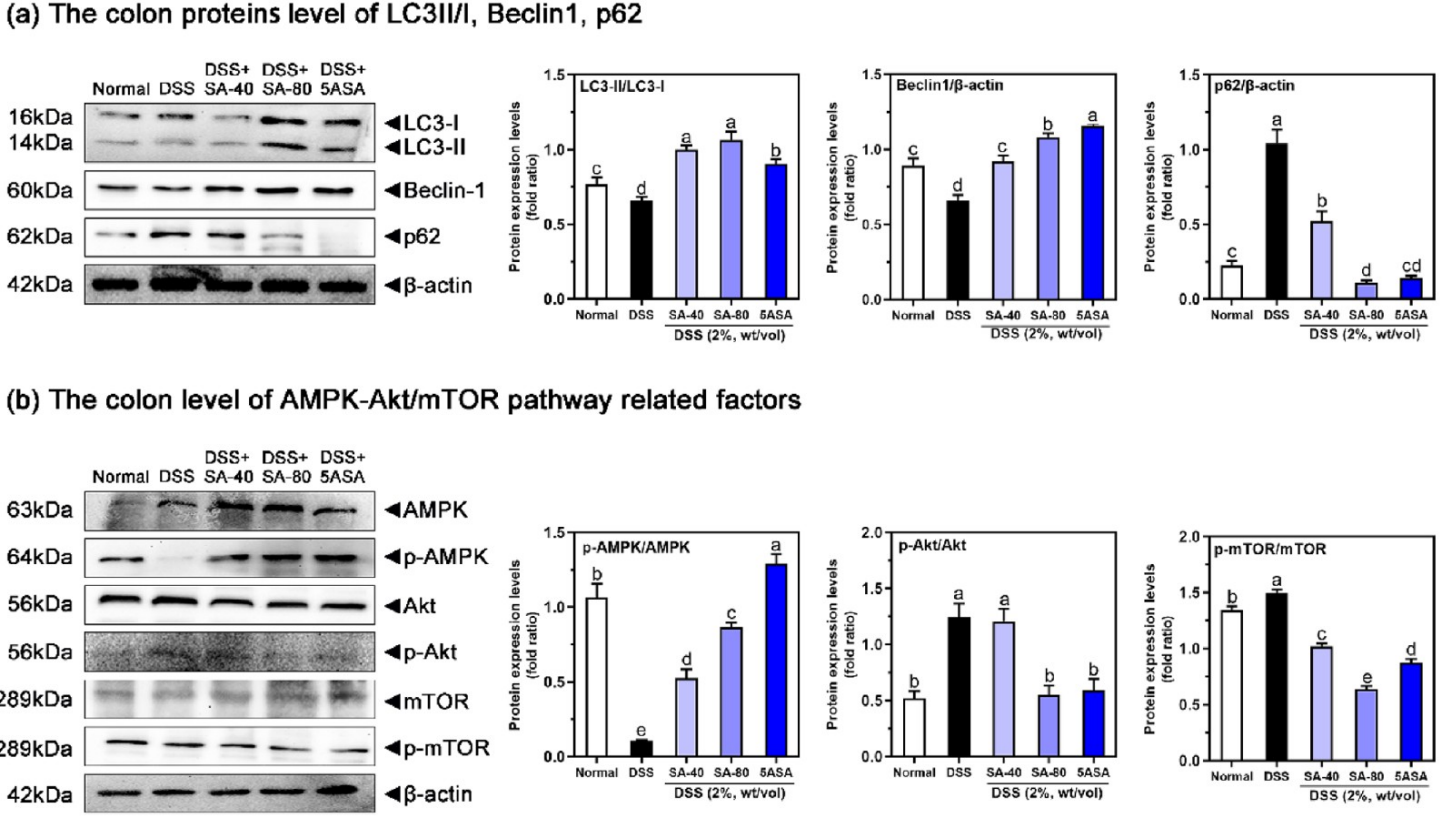
Effects of sinapic acid (SA) on autophagy protein (a) and its related pathway AMPK-Akt/mTOR (b) in DSS-induced chronic colitis mice. Letters above the bar indicate significant differences between the two groups (Duncan’s multiple range test; *p* < 0.05).

The effect of SA on the levels of the autophagy factors p62, LC3-I, LC3-II, and Beclin1 protein in the intestine of mice was examined. DSS-induced mice showed significant reductions in LC3-II/LC3-I (13.72%) and beclin-1 (28%) in the colon, while the 80 mg/kg SA-treated mice showed significant replenishment of the amount of beclin-1 and the ratio of LC3-II to LC3-I (93.5% and 60.5%, *p* < 0.05) (Figure 8(a)). Furthermore, higher protein levels of p62 (∼3.9 times) in the DSS group were observed, and these levels (89.8%) were effectively inhibited in the 80 mg/kg SA group (*p* < 0.05) (Figure 8(a)).

### 3.9 Effects of SA on the AMPK-Akt/mTOR Pathway in Chronic Colitis Mice

To better investigate the mechanism by which SA promotes autophagy, we examined the effect of SA on the AMPK-Akt/mTOR pathway in the colon (Figure 8(b)). DSS-induced chronic colitis increased the p-Akt/Akt and p-mTOR/mTOR ratios (∼1.39 times and 11.51%, *p* < 0.05) and decreased the p-AMPK/AMPK ratio (90.1%, *p* < 0.05) compared to normal animals. After treatment with SA, the AMPK-Akt/mTOR pathway in the colon was improved: 80 mg/kg of SA was more effective, the phosphorylation of Akt (55.9%, *p* < 0.05) and mTOR (57.5%, *p* < 0.05) was inhibited, and phosphorylated AMPK levels (∼7.18 times, *p* < 0.05) were restored.

## 4 Discussion

UC is a chronic disease characterized by repeated inflammation and ulcers, and severe cases can lead to colorectal cancer. Recent studies have suggested that flavonoids and phenolic acids may have beneficial effects in improving colitis.^33^ For instance, salvianolic acid A has a protective effect on colitis mice by inhibiting inflammation and repairing the intestinal mucosa.^34^ Our previous studies have demonstrated that the SA has several biological effects, such as anti-inflammatory, antiobesity, and antitumor effects, and can maintain epithelial homeostasis *in vitro*.^22−24,26^ To investigate the protective effect of SA on colitis as well as its mechanisms, including improvements in intestinal barrier damage, suppressing inflammation, managing oxidative stress, inducing autophagy, sustaining epithelial homeostasis, and alleviating colon fibrosis, we used a chronic colitis mouse model induced by DSS.

Damage to the intestinal mucosal barrier is a major factor in the development of UC. The intestinal mucosal barrier relies on chemical and mechanical barriers to maintain normal physiological functions and improve intestinal permeability.^35^ Mucins produced by intestinal epithelial cells and goblet cells bind to immune cells to form a chemical barrier that stabilizes the contents of the intestinal lumen and mucosa and resists attack by harmful substances.^36^ As our results show, intestinal homeostasis is disrupted when there is a decrease in goblet cells and mucins, and SA supplementation increases the number of goblet cells and the secretion of mucins, thereby reducing the damage associated with intestinal inflammation. Similarly, a mechanical barrier composed of tight junction proteins maintains intestinal mucosal barrier function and regulates intestinal permeability, which in turn prevent exogenous substances from invading the gut.^37^ The findings of this study showed that SA could elevate the expression of Claudin-1, ZO-1, and Occludin proteins, indicating that SA could safeguard the intestinal barrier by controlling both the chemical and mechanical barriers, thereby sustaining the integrity of the intestinal mucosal barrier and enhancing the intestinal permeability.

During the development of UC, DSS penetrates the intestinal mucosa, leading to destruction of intestinal epithelial cells and inflammation of mouse colon tissue.^38,39^ The UC symptoms of NLRP3 gene-deficient mice were alleviated.^40^ Caspase-1 gene-deficient mice significantly reduced their susceptibility to UC by inhibiting the secretion of pro-inflammatory cytokines IL-1β and IL-18.^41^ IL-1β induces the synthesis of inflammatory factors (e.g., IL-6) and acts synergistically with IL-17 and IL-18 to mediate intestinal inflammatory responses.^42,43^ In this study, serum levels of IL-1β, IL-6, TNF-α, IL-17A, and IL-18 were elevated in the DSS-fed mice. Some polyphenolic compounds (such as chlorogenic acid and wogonoside) have been shown to inhibit the NLRP3 inflammasome, thus preventing colitis.^38,44^ In our previous study,^23^ SA could inhibit the NLRP3 cascade reaction and inflammation in DSS-fed mice with colitis. This study further validated the results, demonstrating the importance of preventing the activation of NLRP3 in inhibiting inflammation.

Intestinal epithelial damage and destruction caused by excessive inflammation and oxidative stress are the chief pathological features of UC.^45^ The excess pro-inflammatory cytokines can alter the redox balance in the intestinal mucosa, with consequent oxygen radical overload exacerbating the inflammation and ultimately disrupting the intestinal barrier.^46^ Our present study indicated that SA can restore the abnormality of antioxidant factors in the intestinal mucosa of DSS-induced chronic colitis mice and reduce oxidative damage, which is consistent with our earlier findings.^23^ Here, we also found that SA supplementation increased the phosphorylation of the Nrf2 protein and decreased the expression level of Keap1 in the nucleus. Nrf2 is critical for resistance to ROS and free radical overproduction by dissociating from Keap1 and translocating into the nucleus to bind to the control ARE as a means of inducing the expression of the antioxidant factors heme oxygenase (HO)-1, SOD, GPx, and CAT and thus negatively regulating the pro-inflammatory response that occurs in the intestinal mucosa.^47,48^ Evidence suggests that activation of the Nrf2 pathway is effective against tissue inflammation and mitigates intestinal damage.^37^ By blocking the degradation of Nrf2, the expression of Keap1 is decreased, which in turn increases the levels of HO-1, SOD, and GSH-Px, thereby decreasing intestinal inflammation and oxidative stress.^13^

The inflammatory cytokines promote fibroblast activation and secretion of matrix proteins (such as collagen), which lead to thickening and fibrosis of the intestinal wall tissue in intestinal inflammation.^49^ Oxidative stress can participate in the occurrence of intestinal fibrosis through various pathways, such as promoting the activation and proliferation of fibroblasts and inhibiting matrix metalloproteinases.^50^ When there is a reoccurrence of injury to the intestine, it causes an accumulation of inflammation and an overabundance of collagen in the intestine, as seen in Crohn’s disease patients. In these cases, the submucosal layer of the constricted colonic tissues has increased levels of collagen as well as an increase in fibrosis-associated elements like α-SMA, collagen-I, and fibronectin.^29,51,52^ In this experiment, due to repeated stimulation by DSS, α-SMA and collagen-I proteins increased continuously in colitis mice, resulting in thickening of the intestinal wall. Meanwhile, SA intervention significantly reduced DSS-induced colonic collagen accumulation, inhibited the increase in colonic α-SMA and type I collagen, and reduced intestinal fibrosis. Consistent with this, the antifibrotic effects of polyphenolic compounds, such as curcumin and resveratrol, have been demonstrated in intestinal fibrosis models.^53,54^

Research in recent years has indicated that autophagy may be a potential treatment for fibrotic diseases in multiple organs, such as the lung,^55^ kidney,^56^ and myocardium,^57^ yet investigations into its role in colonic fibrosis are still limited. Studies involving mice have revealed that autophagy inhibitor treatment leads to considerable intestinal fibrosis and a considerable amount of collagen being deposited in the mucosa, submucosa, and subplasma layer.^52^ Autophagy that is insufficient can worsen inflammation, resulting in fibrosis and cancer. However, by restraining the immune-inflammatory reaction and suppressing the formation of the NLRP3 inflammasome in the colon, autophagy can be activated, thus reducing the advancement of intestinal fibrosis.^8^ Potential damage accompanying colonic inflammation and fibrosis is well controlled by autophagy.^58^ Autophagy stimulation enhances collagen degradation to attenuate fibrosis, whereas autophagy inhibition increases pro-inflammatory cytokines and pro-fibrotic factors.^59^ LC3 is a specific marker of autophagosomes, and the ratio of the LC3-II and LC3-I is used to monitor autophagy occurrence.^60^ As a measure of autophagic flux, p62 is negatively correlated to autophagic activity. Beclin1 is a key participant in autophagy and regulates the synthesis and maturation of autophagosomes.^16^ In the TNBS mouse model of chronic colitis, the autophagy indicator LC3-BII/I ratio was reduced, and inflammatory and fibrogenic factors were increased,^58^ promoting intestinal fibrosis.^61^ The present study also found that the expression of Beclin1 and LC3 was increased, and that of p62 was decreased by SA intervention, demonstrating that SA can inhibit the occurrence of chronic colitis and colonic fibrosis by regulating autophagy.

The AMPK-Akt/mTOR pathway is closely related to autophagy. mTOR, which is an upstream factor of autophagy, is a key molecule that can be regulated by multiple signaling molecules to inhibit autophagy.^62^ AMPK is an upstream factor of mTOR that regulates the activation of autophagy by inhibiting mTOR.^63^ Akt is also an upstream factor of mTOR. When Akt is phosphorylated, it initiates autophagy along with mTOR.^64^ We found that SA promoted AMPK phosphorylation and reduced Akt and mTOR phosphorylation in the colon, which induced autophagy. The polyphenolic compound resveratrol reduces inflammatory mediators by inhibiting Akt and mTOR levels in the colonic tissues of rats with colitis, which is consistent with our results.^65^ Another phenolic compound, procyanidin A1, has been reported to alleviate UC by regulating AMPK, mTOR, and related factors to change autophagy in a DSS-induced murine model of ulcerative colitis.^66^

## 5 Conclusion

The present study demonstrated that SA has the ability to enhance intestinal barrier integrity, inhibit the activation of the NLRP3 inflammasome, reduce the amount of pro-inflammatory factors, and increase the activity of antioxidant enzymes to alleviate colitis and is associated with the regulation of the Nrf2/Keap1 pathway. In addition, SA can reduce colitis and intestinal fibrosis by modulating the AMPK-Akt/mTOR pathway, inducing autophagy production, and reducing the production of the fibrotic protein type I collagen and α-SMA. In conclusion, our findings provide new clues for the treatment of colitis and intestinal fibrosis with SA, and these effects will be further confirmed in future clinical trials.
